# Risk factors for postoperative complications in laparoscopic and robot‐assisted surgery for octogenarians with colorectal cancer: A multicenter retrospective study

**DOI:** 10.1002/ags3.12874

**Published:** 2024-12-04

**Authors:** Takehito Yamamoto, Koya Hida, Kentaro Goto, Meiki Fukuda, Susumu Inamoto, Hiroki Hashida, Ryo Matsusue, Ryo Takahashi, Rei Mizuno, Hiroaki Terajima, Kazutaka Obama

**Affiliations:** ^1^ Department of Surgery, Graduate School of Medicine Kyoto University Kyoto Japan; ^2^ Department of Gastroenterological Surgery and Oncology Medical Research Institute Kitano Hospital Osaka Japan; ^3^ Department of Surgery Japanese Red Cross Osaka Hospital Osaka Japan; ^4^ Department of Surgery Kobe City Medical Center General Hospital Hyogo Japan; ^5^ Department of Gastrointestinal Surgery Tenri Yorozu Hospital Nara Japan; ^6^ Department of Surgery Kyoto Katsura Hospital Kyoto Japan; ^7^ Department of Surgery Kyoto Medical Center Kyoto Japan

**Keywords:** Japan, laparoscopy, postoperative complications, risk factors, robotic surgical procedures

## Abstract

**Aim:**

Minimally invasive surgery for colorectal cancer is increasing globally. However, the safety in older patients have not been thoroughly examined.

**Methods:**

Patients with colorectal cancer who underwent laparoscopic or robot‐assisted surgery at Kyoto University Hospital and 18 affiliated institutions in Japan that participated in the Kyoto Colorectal Surgery Group between 2018 and 2023 were enrolled. Focusing on patients ≥80 y, we investigated the risk factors for postoperative complications.

**Results:**

In total, 7303 patients were enrolled in this study. The mean age was 71 ± 11 y, with 1665 patients (22.8%) ≥80 y old. Older patients (≥80 y) had significantly higher ASA and ECOG‐PS scores and more comorbidities including diabetes, chronic obstructive pulmonary disease (COPD), hypertension, heart disease, and cerebrovascular disease than patients ≤79 y old (all *p* < 0.05). In the older group, postoperative complications (Clavien–Dindo grade ≥II) occurred in 210 patients (12.6%). After adjusting for covariates using the multivariable logistic regression model, rectal cancer (odds ratio [OR]: 1.84, 95% confidence interval [CI]: 1.30–2.60, *p* = 0.001), operation time ≥300 min (OR: 1.52, 95% CI: 1.07–2.16, *p* = 0.020), and blood loss ≥100 mL (OR: 2.19, 95% CI: 1.80–3.24, *p* < 0.001) were associated with the occurrence of complications, whereas their comorbidities showed no association.

**Conclusion:**

In minimally invasive colorectal cancer surgery for older patients (≥80 y old), prioritizing shorter operation time and blood loss control is crucial, especially for patients with rectal cancer because of their high risk of complications.

## Graphical abstract

Patients with colorectal cancer who underwent laparoscopic or robotic surgery at 19 institutions in Japan between 2018 and 2023 were enrolled. Focusing on patients ≥80 y, we investigated the risk factors for postoperative complications. As a result, rectal cancer, operation time ≥300 min and blood loss ≥100 mL were associated with the occurrence of complications, whereas their comorbidities showed no association.

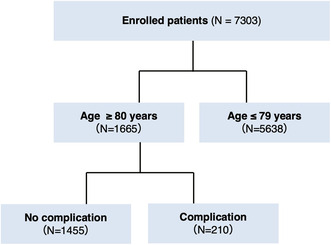

## 1 INTRODUCTION

Population aging is advancing primarily in developed countries, leading to an increase in the number of surgeries performed on older patients with colorectal cancer. Recent technical advancements have improved short‐term mortality in octogenarians,^1^ whereas prior studies have consistently shown that older patients have a higher risk of complications following colorectal surgery than younger patients.^2^, ^3^, ^4^, ^5^ In particular, nonsurgical systemic complications are notably more prevalent in the older population compared with the younger population,^6^, ^7^ probably owing to the higher burden of comorbidities in older patients. Furthermore, once complications arise, they tend to escalate in severity and can lead to adverse outcomes, including mortality.

Previous studies have evaluated the surgical risk for older patients with colorectal cancer; however, few have focused on laparoscopic or robot‐assisted surgery. These minimally invasive techniques have the potential to reduce the physical burden on older patients.^8^, ^9^

The use of minimally invasive surgery in patients with colorectal cancer is increasing globally. More than 80% of the colorectal cancer surgeries in Japan are minimally invasive.^10^ Japan, renowned for its longevity, boasts an average life expectancy of 81 y for males and 87 y for females. This demographic trend has led to a considerable increase in the prevalence of older patients with colorectal cancer. In a recent year, Japan recorded over 160 000 new cases of colorectal cancer, with >30% of these cases occurring in individuals ≥80 y old.^11^ Therefore, we emphasize the importance of disseminating evidence on the safety of minimally invasive surgery among older patients, particularly from Japan.

This study aimed to investigate the risk factors for postoperative complications in patients ≥80 y old following laparoscopic and robot‐assisted surgery for colorectal cancer, and to propose preventative strategies.

## 2 METHODS

### 2.1 Study design and patients

This multicenter, retrospective study enrolled consecutive patients with colorectal cancer who underwent laparoscopic or robot‐assisted surgery at Kyoto University Hospital and 18 affiliated institutions in Japan that participated in the Kyoto Colorectal Surgery Group between 2018 and 2023. Included were patients with colorectal malignancies whose primary lesions were resected, including adenocarcinoma, neuroendocrine tumors, and gastrointestinal stromal tumors, whereas those who solely underwent stoma creation without primary lesion resection were excluded.

This study was approved by the Kyoto University Graduate School and Faculty of Medicine Ethics Committee (reference no. R0286‐7), and from each participating institution. The study design and article preparation conform to the Strengthening the Reporting of Observational Studies in Epidemiology (STROBE) reporting guideline.

### 2.2 Data collection

Cohort data were collected using REDCap software (Vanderbilt University). Patient characteristics included age, sex, body mass index (BMI), American Society of Anesthesiologists (ASA) scores, Eastern Collaborative Oncology Group performance status (ECOG‐PS), smoking history within 1 year, and comorbidities such as diabetes mellitus, hypertension, chronic obstructive pulmonary disease (COPD), heart diseases (coronary heart disease and heart failure), dialysis owing to chronic kidney disease, cerebrovascular disease, and corticosteroids use (regular oral or intravenous corticosteroid administration for chronic medical conditions within 30 d before surgery). Additionally, operative and oncological information was collected, including the primary tumor location (colon or rectum), tumor markers, UICC TNM classification, operative approach, operation time, and blood loss.

### 2.3 Study outcomes

Study participants were divided into two groups based on age at the time of operation: an older group (≥80 y) and a younger group (≤79 y), and their clinical characteristics were compared. The investigation focused on the older group, aiming to identify risk factors for postoperative complications, defined as a Clavien–Dindo classifications grade ≥II.^12^

### 2.4 Statistical analyses

Continuous variables are presented as the median (interquartile range [IQR]) or mean ± standard deviation (SD), whereas categorical variables are presented as absolute numbers with percentages. The chi‐square or Fisher's exact test were used to compare categorical variables (Fisher's exact test was applied to factors with cells containing fewer than five data points). The Wilcoxon rank‐sum test or Student's *t*‐test were used to compare continuous variables. Cutoff values for operation time and blood loss were determined based on the IQR and clinical knowledge. Multivariate logistic regression analysis, considering potential confounders identified from the previous literature and clinical knowledge (including age, sex, BMI, ASA score, ECOG‐PS, comorbidities, smoking status, main tumor location, TNM classification, operative approach, operation time, and blood loss), was used to evaluate the correlation between patient characteristics and complications.^2^, ^3^, ^4^, ^5^, ^13^, ^14^, ^15^, ^16^, ^17^, ^18^, ^19^, ^20^ The associations are expressed as point estimates of odds ratios (ORs), 95% confidence intervals (CIs), and *p* values. Statistical analyses were performed using JMP Pro, v. 17 (SAS Institute, Cary, NC, USA).

## 3 RESULTS

### 3.1 Patient characteristics

**FIGURE 1:**
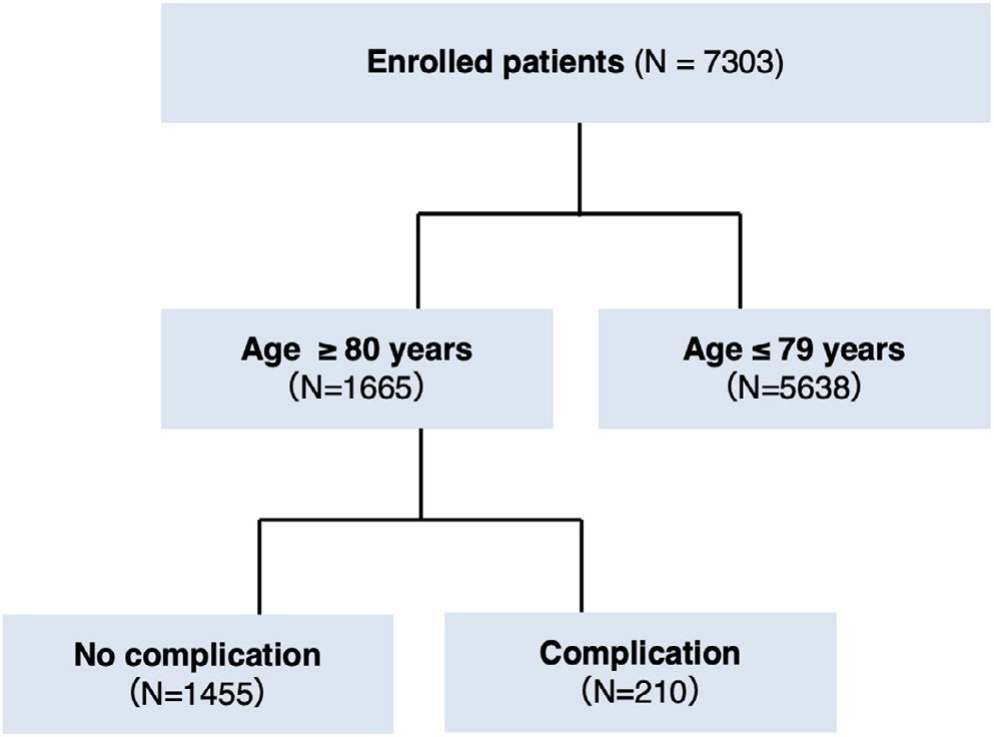
The flowchart of the study population.

**FIGURE 2:**
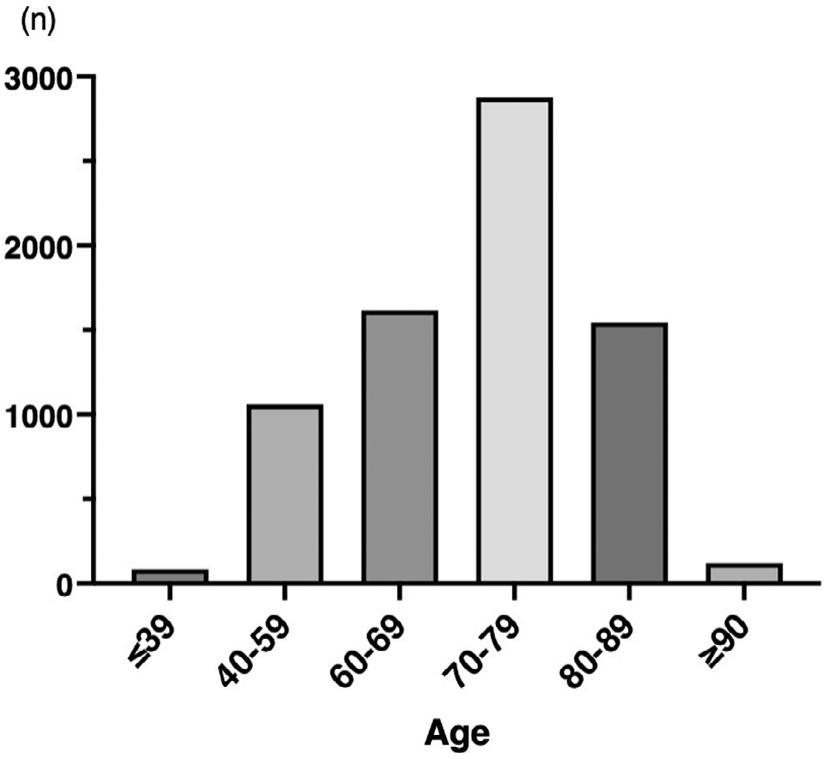
Age distribution of the study population.

As shown in Figure 1, 7303 patients were enrolled. The age distribution of the study population is shown in Figure 2. Of the total, 1665 patients (22.8%) were ≥80 y old, whereas 5638 patients were ≤79 y old. The clinical characteristics of all study participants and a comparison between the older (≥80 y) and younger patients (≤79 y) are presented in Table 1. The mean age of all patients was 71 ± 11 y with 4088 patients (56.0%) being male. Among the cohort, 4707 patients (64.5%) were diagnosed with colon cancer, whereas the remaining 2596 patients (35.5%) had rectal cancer. All patients underwent minimally invasive surgery; 6992 patients (95.7%) underwent laparoscopic surgery, whereas the remaining 311 patients (4.3%) underwent robot‐assisted surgery.

**TABLE 1:** Comparison of clinical characteristics between older patients (≥80 y old) and younger patients (≤79 y old).

	All participants (*n* = 7303)	Comparisons		
Variables		Older patients ≥80 y old (*n* = 1665)	Younger patients ≤79 y old (*n* = 5638)	*p* Value
Age (y)	71 ± 11	84 ± 3	67 ± 10	<0.001*
Sex				<0.001*
Male	4088 (56.0)	803 (48.2)	3285 (58.3)	
Female	3215 (44.0)	862 (51.8)	2353 (41.7)	
BMI (kg/m^2^)	22.5 ± 3.7	22.1 ± 3.3	22.6 ± 3.7	<0.001*
ASA score				<0.001*
1–2	6319 (86.5)	1306 (78.4)	5013 (88.9)	
3–4	980 (13.4)	357 (21.4)	623 (11.1)	
ECOG‐PS				<0.001*
0–1	6515 (89.2)	1301 (78.1)	5214 (92.5)	
≥2	562 (7.7)	305 (18.3)	257 (4.6)	
Smoking	1265 (17.3)	94 (5.2)	1171 (20.8)	<0.001*
Steroid use	149 (2.0)	31 (1.9)	118 (2.1)	0.560
Comorbidity				
Diabetes	1422 (19.5)	356 (21.4)	1066 (18.9)	0.024*
COPD	362 (5.0)	113 (6.8)	249 (4.4)	<0.001*
Hypertension	3244 (44.4)	982 (59.0)	2262 (40.1)	<0.001*
Heart disease	246 (3.4)	97 (5.8)	149 (2.6)	<0.001*
Dialysis	89 (1.2)	20 (1.2)	69 (1.2)	0.943
Cerebrovascular disease	432 (5.9)	157 (9.4)	275 (4.9)	<0.001*
Tumor location				<0.001
Colon	4707 (64.5)	1238 (74.4)	3469 (61.5)	
Rectum	2596 (35.5)	427 (25.6)	2169 (38.5)	
CEA >5 ng/mL	802 (11.0)	204 (12.2)	598 (10.6)	<0.001*
cT classification				<0.001*
cT0–2	2319 (31.8)	442 (26.5)	1877 (33.3)	
cT3–4	4917 (67.3)	1208 (72.6)	3709 (65.8)	
cN classification				0.722
cN0	4076 (55.8)	919 (55.2)	3157 (56.0)	
cN1‐3	3144 (43.0)	720 (43.2)	2424 (43.0)	
cStage				<0.001*
0	55 (0.8)	9 (0.5)	46 (0.8)	
I	2016 (27.6)	386 (23.1)	1630 (28.9)	
II	1826 (25.0)	488 (29.3)	1338 (23.7)	
III	2495 (34.2)	597 (35.9)	1898 (33.7)	
IV	767 (10.5)	148 (8.9)	619 (11.0)	
Operative approach				0.002*
Laparoscopy	6992 (95.7)	1617 (97.1)	5375 (95.3)	
Robot	311 (4.3)	48 (2.9)	263 (4.7)	
Operation time (min)	275 [216–357]	259 [176–328]	282 [219–366]	<0.001*
Blood loss (mL)	7 [0–45]	7 [0–47]	8 [0–45]	0.766

*Note*: Results are presented as mean ± SD, median [IQR], or *n* (%).

Abbreviations: ASA score, American Society of Anesthesiologists score; BMI, body mass index; CEA, carcinoembryonic antigen; COPD, chronic obstructive pulmonary disease; ECOG‐PS, Eastern Cooperative Oncology Group performance status; IQR, Interquartile range.

* *p* <0.05

In the ≥80 y group, ASA and ECOG‐PS scores were significantly higher, and the prevalence of almost all the comorbidities including diabetes, COPD, hypertension, heart disease, and cerebrovascular disease were significantly higher than that in patients ≤79 y (all *p* < 0.05).

### 3.2 Risk factors for postoperative complications

Among all the study participants, postoperative complications were observed in 801 patients (11.0%). Those experiencing complications had a significantly longer postoperative hospital stay than those without complications (21 d vs. 10 d [median], *p* < 0.001). Among those aged ≥80 y, 210 patients (12.6%) experienced postoperative complications, whereas among those ≤79 y old, 591 patients (10.5%) experienced postoperative complications.

Postoperative complications are outlined in Table 2. In the older group, the most frequently observed complications were ileus (*n* = 77, 4.6%), followed by pneumonia (*n* = 37, 2.2%), and urinary infection (*n* = 29, 1.7%), while in the younger group, the most frequently observed complications were ileus (*n* = 200, 3.5%), followed by anastomotic leakage (*n* = 160, 2.8%) and urinary disorder (*n* = 86, 1.5%). We found that the incidence of systemic complications, including pneumonia, pulmonary embolism, deep vein thrombosis, heart failure, and renal failure, was significantly higher in the older group compared to the younger group. Furthermore, the incidence of ileus was also significantly higher in the older group than in the younger group. Conversely, the incidence of anastomotic leakage was significantly higher in the younger group compared to the older group.

**TABLE 2:** Postoperative complications (Clavien–Dindo Classification grade ≥II) in the older patients (age ≥80 y old) and younger patients (≤79 y old).

	Older patients ≥80 y old (*n* = 1665)	Younger patients ≤79 y old (*n* = 5638)	*p* Value
Complications	210 (12.6)	591 (10.5)	0.015*
Ileus	77 (4.6)	200 (3.5)	0.043*
Pneumonia	37 (2.2)	35 (0.6)	<0.001*
Urinary infection	29 (1.7)	83 (1.5)	0.447
Anastomotic leakage	27 (1.6)	160 (2.8)	0.005*
Urinary disorder	23 (1.4)	86 (1.5)	0.670
Wound infection	20 (1.3)	77 (1.4)	0.723
Gastrointestinal bleeding	16 (1.2)	32 (0.6)	0.081
Pulmonary embolism	9 (0.5)	8 (0.1)	0.003*
Deep vein thrombosis	9 (0.5)	11 (0.2)	0.018*
Heart failure	8 (0.5)	3 (0.1)	<0.001*
Central nerve system disorder	8 (0.5)	14 (0.2)	0.132
Renal failure	6 (0.4)	5 (0.1)	0.023*
Intra‐abdominal bleeding	5 (0.3)	12 (0.2)	0.562

*Note*: Results are presented as *n* (%).

* *p* <0.05

**FIGURE 3:**
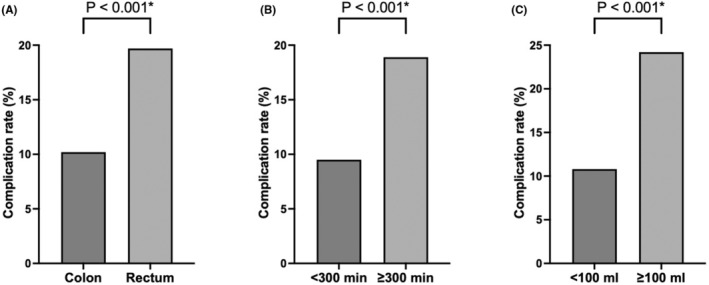
Comparison of the complication rates between patients with and without each risk factor: (A) tumor location, (B) operation time, (C) blood loss. *p <0.001

Table 3 presents the results of the univariate and multivariate logistic regression model after adjusting for covariates in the older group. Rectal cancer (OR: 1.84, 95% CI: 1.30–2.60, *p* = 0.001), operation time ≥300 min (OR: 1.52, 95% CI: 1.07–2.16, *p* = 0.020), and blood loss ≥100 mL (OR: 2.19, 95% CI: 1.80–3.24, *p* < 0.001) were associated with the occurrence of complications. In addition, age ≥90 y showed a trend toward association with the occurrence of postoperative complications (OR: 1.64, 95% CI: 0.96–2.80, *p* = 0.072). Figure 3 shows the comparison of complication rates for patients with and without each specific risk factor.

**TABLE 3:** Univariate and multivariate analysis for risk factors of postoperative complications in the older group (age ≥80 y old).

			Univariate analysis			Multivariate analysis		
Variables	No complication (*n* = 1455)	Complication (*n* = 210)	OR	95%CI	*p* Value	OR	95%CI	*p* Value
Age (y)								
≤89	1359 (93.4)	186 (88.6)						
≥90	96 (6.6)	24 (11.4)	1.83	1.14–2.93	0.011*	1.64	0.96–2.80	0.072
Sex								
Male	687 (47.2)	116 (55.2)	1.38	1.03–1.85	0.030*	1.31	0.95–1.81	0.099
Female	768 (52.8)	94 (44.8)						
BMI (kg/m^2^)								
<25	1196 (82.2)	171 (81.4)						
≥25	259 (17.8)	39 (18.6)	1.05	0.73–1.53	0.785	0.98	0.66–1.45	0.907
ASA score								
1–2	1153 (79.2)	153 (72.9)						
3–4	300 (20.6)	57 (27.1)	1.43	1.03–1.99	0.032*	1.24	0.84–1.84	0.269
ECOG‐PS								
0–1	1143 (78.6)	158 (75.2)						
≥2	259 (17.8)	46 (21.9)	1.28	0.90–1.83	0.166	1.20	0.81–1.79	0.362
Smoking	85 (5.8)	9 (4.3)	0.71	0.35–1.44	0.341	0.58	0.27–1.27	0.174
Steroid use	27 (1.9)	4 (1.9)	1.00	0.35–2.88	1.000	1.16	0.39–3.43	0.792
Diabetes	313 (21.5)	43 (20.5)	0.94	0.65–1.34	0.714	0.85	0.58–1.25	0.399
COPD	94 (6.5)	19 (9.0)	1.42	0.85–2.38	0.180	1.57	0.87–2.82	0.132
Hypertension	853 (58.6)	129 (61.4)	1.08	0.80–1.46	0.607	0.97	0.71–1.33	0.852
Heart disease	83 (5.7)	14 (6.7)	1.14	0.64–2.06	0.651	0.96	0.51–1.84	0.913
Dialysis	16 (1.1)	4 (1.9)	1.74	0.58–5.25	0.307	1.55	0.48–4.96	0.461
Cerebrovascular disease	130 (8.9)	27 (12.9)	1.46	0.94–2.27	0.094	1.46	0.91–2.34	0.115
Tumor location								
Colon	1112 (76.4)	126 (60.0)						
Rectum	343 (23.6)	84 (40.0)	2.16	1.60–2.92	<0.001*	1.84	1.30–2.60	0.001*
CEA ≥5 ng/mL	184 (12.6)	20 (9.5)	0.73	0.45–1.18	0.197	0.62	0.36–1.06	0.081
cT classification								
cT1–2	383 (26.3)	59 (28.1)						
cT3–4	1058 (72.7)	150 (71.4)	0.92	0.67–1.27	0.615	0.91	0.62–1.33	0.629
cN classification								
cN0	803 (55.2)	116 (55.2)						
cN1‐3	627 (43.1)	93 (44.3)	1.03	0.77–1.38	0.859	1.04	0.74–1.47	0.820
Distant metastasis	130 (8.9)	18 (8.6)	0.93	0.55–1.56	0.779	1.02	0.56–1.84	0.959
Operative approach								
Laparoscopy	1420 (97.6)	197 (93.8)						
Robot	35 (2.4)	13 (6.2)	2.68	1.39–5.15	0.002*	1.32	0.65–2.69	0.440
Operation time (min)								
<300	1003 (68.9)	105 (50.0)						
≥300	451 (31.0)	105 (50.0)	2.22	1.66–2.98	<0.001*	1.52	1.07–2.16	0.020*
Blood loss (ml)								
<100	1283 (88.2)	155 (73.8)						
≥100	172 (11.8)	55 (26.2)	2.65	1.87–3.74	<0.001*	2.19	1.80–3.24	<0.001*

*Note*: Results are presented as *n* (%).

Abbreviations: ASA score, American Society of Anesthesiologists score; BMI, body mass index; CEA, carcinoembryonic antigen; CI, confidence interval; COPD, chronic obstructive pulmonary disease; ECOG‐PS, Eastern Cooperative Oncology Group performance status; OR, odd ratio.

* *p* <0.05

## 4 DISCUSSION

To our knowledge, the present study is one of the largest investigations focusing on patients with colorectal cancer aged 80 y and older who underwent minimally invasive surgery. Notably, many clinical trials tend to exclude older patients, particularly those aged 80 and older, rendering the findings of this multicenter collaborative study valuable.

We first compared the characteristics of patients with colorectal cancer aged ≥80 y with those aged ≤79 y. As anticipated, older patients exhibited higher ASA and ECOG‐PS scores, and more comorbidities than younger patients. Despite these seemingly vulnerable features, neither the ASA score nor ECOG‐PS independently affected the occurrence of complications after minimally invasive surgery.

These comparisons also indicated that significantly more patients with higher CEA and greater cT classification were found in the older group than in the younger group. The reason for this may be that many older individuals do not undergo screenings, leading to the discovery of colorectal cancer at a more advanced stage. In addition, there were significantly more cases of robot‐assisted surgery in younger patients. This can be explained by the fact that, in the early phase of introducing robot‐assisted surgery, priority was given to younger patients over high‐risk older patients. Furthermore, the operation time was significantly longer in younger patients. This may be due to the fact that longer operations tended to be avoided on older patients compared with younger patients.

As shown in Table 2, the incidence of systemic complications was significantly higher in the older group compared to the younger group. Considering the frailty of physical function in elderly patients, this result is not unexpected and is consistent with the previous reports.^6^, ^7^ It underscores the necessity of careful treatment planning for older individuals, with particular attention to the increased risk of systemic complications. In addition, postoperative ileus was observed more frequently in the older group. This finding aligns with a previous large‐scale meta‐analysis of nearly 30 000 patients undergoing colorectal surgery, which identified advanced age as a significant risk factor for postoperative ileus.^21^ The elevated risk in older patients appears to be attributable to age‐related declines in gastrointestinal function and reduced postoperative mobility. Conversely, the incidence of anastomotic leakage was significantly higher in the younger group compared to the older group. The interpretation of this result is challenging; however, it is presumed to be primarily attributable to the significantly higher proportion of rectal cancer patients in the younger group than in the older group, as demonstrated in Table 1 (25.6% vs. 38.5%, *p* < 0.001).

Table S1 shows the results of the univariate and multivariate analyses for risk factors of complications, respectively, in the younger group. The risk factors for postoperative complications were similar between the older and younger group: rectal cancer, longer operation time, and larger amount of blood loss. We considered that this may be attributed to the minimally invasive nature of laparoscopic and robot‐assisted surgery for older patients. Notably, male sex was the only risk factor present in the younger group but not in the older group. As shown in Table 1, the number of male patients was significantly smaller in the older group than in the younger group (*p* < 0.001) and the gender ratio reversed with increasing age, which reflects the fact that females have a longer average lifespan than males. This may mean that healthier males were selected in the older group and, therefore, male sex was not a risk factor in the older group.

The previous reports on the risk factors for complications after colorectal cancer surgery in older patients have indicated the negative impact of patient comorbidities and ASA score on postoperative outcomes.^13^, ^14^, ^15^ For example, a population‐based study by Niemelainen et al that enrolled 386 patients with colon cancer aged ≥80 y who underwent curative surgery showed that a higher ASA score and Charlson Comorbidity Index could influence overall survival.^14^ This discrepancy in findings between their studies and the present one may also be attributed to the minimally invasive nature of laparoscopic and robot‐assisted surgery, providing a safer intervention for older patients with vulnerable features.

The present study indicated a negative effect of increased blood loss on the short‐term outcomes of colorectal cancer surgery in older patients. Okamura et al reported in their large‐scale multicenter study that blood loss ≥200 mL is an independent risk factor for postoperative complications in older patients.^19^ Although a considerable portion of the enrolled patients (64%) in that study underwent open surgery, this result is consistent with ours, indicating that the risk of complications resulting from the physical burden of blood loss in older patients cannot be mitigated, even with the use of a minimally invasive approach. Furthermore, the present study indicated that a longer operation time was a risk factor for postoperative complications. A recent large multicenter study including more than 20 000 patients, which evaluated the impact of prolonged operation time in colorectal surgery on short‐term outcomes, revealed that the relative risk of a longer operation time was almost the same between minimally invasive and open surgeries.^22^

Several studies have indicated the superiority of laparoscopic surgery over open surgery for short‐term outcomes in older patients with colorectal cancer.^20^, ^23^, ^24^, ^25^, ^26^ Poles et al investigated 3779 patients with colon cancer older than 85 y old (20.1% underwent minimally invasive surgery), demonstrating a short‐term survival benefit with minimally invasive surgery over open surgery.^20^ Similarly, Chuang et al, analyzing over 40 000 patients aged 80 and over found a significantly lower complication rate with laparoscopic surgery compared to open surgery.^26^ Furthermore, laparoscopic surgery for older patients with colorectal cancer was associated with lower costs and shorter hospital stays.^23^ In contrast, evidence regarding the safety of robot‐assisted surgery for older patients remains limited; however, a few previous studies have suggested potential benefits.^27^, ^28^, ^29^

Few studies have evaluated the surgical outcomes of super‐older patients with colorectal cancer aged 90 y or older, with small participant numbers (*n* = 50–151).^13^, ^30^, ^31^ Furthermore, these studies did not focus on minimally invasive surgery, leaving insufficient evidence in colorectal surgery for nonagenarians. The present study indicated that patients aged 90 and older tend to develop complications compared with octogenarians, emphasizing the need for careful attention to complication risks in nonagenarian patients.

Discussions regarding surgeries for older patients must consider the complications and address the subsequent decline in quality of life (QOL). The GOSAFE (Geriatric Oncology Surgical Assessment and Functional rEcovery after Surgery) study, including 625 patients with colorectal cancer aged of 70 y and older, showed that postoperative complications were significant risk factors for the decline in QOL and failure to achieve functional recovery in patients with colon cancer.^32^ Conversely, a population‐based study by Behman et al, which included 16 479 patients with colorectal cancer ≥70 y old, revealed that minimally invasive surgery decreased physical functional dependency and reduced the need for homecare compared with open surgery.^33^ Also in the present cohort, evaluation of postoperative long‐term outcomes warrants future investigation.

The present study had several limitations. First, pre‐ and postoperative management protocols may vary among institutions, potentially leading to inconsistencies. Prehabilitation could improve postoperative outcomes in older patients with colorectal cancer.^34^ Additionally, the implementation of Enhanced Recovery After Surgery protocols is a crucial strategy for reducing postoperative complication rates in older patients.^35^ In the present cohort, it is possible that various preventive measures for postoperative complications were implemented across different institutions, potentially introducing bias. Second, other factors not included in our analysis, such as preoperative frailty, nutritional status, and muscle volume, may have influenced eventual outcomes. Specifically, preoperative frailty has been identified as an important risk factor for patients with colorectal cancer aged 80 y and above.^36^ Finally, there were 0 to 3.5% missing values per item in our cohort, with a total of 110 missing cases (6.6%) in the older group. This is an important limitation, although this is a figure commonly observed in studies of this scale.

Despite these limitations, we believe that this study will serve as a notable source of information for colorectal surgeons performing minimally invasive surgeries in older patients.

## 5 CONCLUSION

In older patients with colorectal cancer aged 80 and above, complications following minimally invasive surgery were not correlated with patient comorbidities. Instead, significant correlations were observed with rectal cancer, longer operation duration, and increased blood loss. Surgeons should prioritize shortening operation time and controlling blood loss to prevention of complications, particularly in patients with rectal cancer who face a higher risk.

## AUTHOR CONTRIBUTIONS

**Takehito Yamamoto:** Conceptualization; data curation; methodology; writing – original draft. **Koya Hida:** Conceptualization; methodology; writing – review and editing. **Kentaro Goto:** Conceptualization; data curation; project administration; writing – review and editing. **Meiki Fukuda:** Conceptualization; methodology; writing – review and editing. **Susumu Inamoto:** Data curation; project administration; writing – review and editing. **Hiroki Hashida:** Data curation; project administration; writing – review and editing. **Ryo Matsusue:** Conceptualization; project administration; writing – review and editing. **Ryo Takahashi:** Data curation; project administration; writing – review and editing. **Rei Mizuno:** Data curation; project administration; writing – review and editing. **Hiroaki Terajima:** Project administration; supervision; writing – review and editing. **Kazutaka Obama:** Data curation; project administration; supervision; writing – review and editing.

## FUNDING INFORMATION

None.

## CONFLICT OF INTEREST STATEMENT

The authors declare no conflicts of interest for this article.

## ETHICS STATEMENT

Approval of the research protocol by an Institutional Reviewer Board: The study protocol was approved by the Institutional Review Board of Kyoto University (reference no. R0286‐7).

Informed Consent: Informed consent was obtained via an opt‐out form on the website. Patients who declined to participate were excluded.

Registry and the Registration No. of the study/trial: N/A.

Animal Studies: N/A.

## Supporting information


Table S1.
