# Supplementary material for: Risk factors for postoperative complications in laparoscopic and robot‐assisted surgery for octogenarians with colorectal cancer: A multicenter retrospective study
Source: Ann Gastroenterol Surg. 2024 Dec 4;9(2):319–28. doi: 10.1002/ags3.12874 (PMC11877346; doi:10.1002/ags3.12874)
Supplement: Supplementary file 1 — Table S1. [file AGS3-9-319-s001.docx]

**Supplementary Table 1.** Univariate and Multivariate analysis for risk factors of postoperative complications in the younger group (age ≤79 years old)

|  |  |  | Univariate analysis | | | Multivariate analysis | | |
| --- | --- | --- | --- | --- | --- | --- | --- | --- |
| Variables | No complication (n=5047) | Complication (n=591) | OR | 95%CI | P value | OR | 95%CI | P value |
| Age |  |  |  |  |  |  |  |  |
| ≤69 | 2474 (49.0) | 288 (48.7) |  |  |  |  |  |  |
| ≥70 | 2573 (51.0) | 303 (51.3) | 1.01 | 0.85-1.20 | 0.895 | 1.09 | 0.90-1.32 | 0.358 |
| Sex |  |  |  |  |  |  |  |  |
| Male | 2857 (56.6) | 428 (72.4) | 2.01 | 1.67-2.43 | <0.001* | 1.60 | 1.31-1.96 | <0.001* |
| Female | 2190 (43.4) | 163 (27.6) |  |  |  |  |  |  |
| BMI | 22.6±3.7 | 22.6±3.8 |  |  |  |  |  |  |
| <25 | 3881 (76.9) | 462 (78.2) |  |  |  |  |  |  |
| ≥25 | 1163 (23.0) | 128 (21.7) | 0.92 | 0.75-1.14 | 0.456 | 0.86 | 0.69-1.07 | 0.175 |
| ASA score |  |  |  |  |  |  |  |  |
| 1-2 | 4506 (89.3) | 507 (85.8) |  |  |  |  |  |  |
| 3-4 | 539 (10.7) | 84 (14.2) | 1.39 | 1.08-1.77 | 0.010* | 1.45 | 0.97-2.18 | 0.069 |
| ECOG-PS |  |  |  |  |  |  |  |  |
| 0-1 | 4669 (92.5) | 545 (92.2) |  |  |  |  |  |  |
| ≥ 2 | 215 (4.3) | 42 (7.1) | 1.67 | 1.19-2.36 | 0.003* | 1.45 | 0.96-2.20 | 0.075 |
| Smoking | 1015 (20.1) | 156 (2.6) | 1.40 | 1.15-1.70 | 0.001* | 1.07 | 0.86-1.33 | 0.527 |
| Steroid use | 102 (2.0) | 16(2.7) | 1.31 | 0.77-2.24 | 0.320 | 1.37 | 0.77-2.46 | 0.287 |
| Diabetes | 946 (18.7) | 120 (20.3) | 1.10 | 0.89-1.36 | 0.372 | 1.02 | 0.81-1.29 | 0.855 |
| COPD | 214 (4.2) | 35 (5.9) | 1.40 | 0.97-2.03 | 0.069 | 1.15 | 0.77-1.71 | 0.507 |
| Hypertension | 2020 (40.0) | 242 (40.9) | 1.02 | 0.85-1.21 | 0.860 | 0.99 | 0.82-1.21 | 0.947 |
| Heart disease | 138 (2.7) | 11 (1.9) | 0.66 | 0.35-1.22 | 0.179 | 0.54 | 0.28-1.02 | 0.059 |
| Dialysis | 63 (1.2) | 6 (1.0) | 0.81 | 0.35-1.88 | 0.620 | 0.72 | 0.30-1.76 | 0.473 |
| Cerebrovascular disease | 236 (4.7) | 39 (6.6) | 1.40 | 0.99-1.98 | 0.059 | 1.13 | 0.77-1.67 | 0.536 |
| Tumor location |  |  |  |  |  |  |  |  |
| Colon | 3243 (64.3) | 226 (38.2) |  |  |  |  |  |  |
| Rectum | 1804 (35.7) | 365 (61.8) | 2.90 | 2.44-3.46 | < 0.001* | 2.23 | 1.83-2.71 | <0.001* |
| CEA ≥ 5 | 519 (10.3) | 79 (13.4) | 1.35 | 1.04-1.73 | 0.022* | 1.08 | 0.81-1.44 | 0.588 |
| cT classification |  |  |  |  |  |  |  |  |
| cT1–2 | 1720 (34.1) | 157 (26.6) |  |  |  |  |  |  |
| cT3–4 | 3278 (64.9) | 431 (72.9) | 1.44 | 1.19-1.74 | <0.001* | 1.11 | 0.88-1.40 | 0.380 |
| cN classification |  |  |  |  |  |  |  |  |
| cN0 | 2881 (57.1) | 276 (46.7) |  |  |  |  |  |  |
| cN1-3 | 2112 (41.8) | 312 (52.8) | 1.54 | 1.30-1.83 | <0.001* | 1.18 | 0.95-1.46 | 0.128 |
| Distant metastasis | 536 (10.6) | 83 (14.0) | 1.35 | 1.05-1.73 | 0.019* | 1.04 | 0.78-1.39 | 0.774 |
| Operative approach |  |  |  |  |  |  |  |  |
| Laparoscopy | 4844 (96.0) | 531 (89.8) |  |  |  |  |  |  |
| Robot | 203 (4.0) | 60 (10.2) | 2.70 | 1.99-3.64 | <0.001* | 1.26 | 0.91-1.75 | 0.156 |
| Operation time |  |  |  |  |  |  |  |  |
| <300 | 2988 (59.2) | 193 (32.6) |  |  |  |  |  |  |
| ≥300 | 2049 (40.6) | 398 (67.3) | 3.02 | 2.52-3.62 | <0.001* | 1.84 | 1.50-2.27 | <0.001* |
| Blood loss |  |  |  |  |  |  |  |  |
| <100 | 4433 (87.8) | 437 (73.9) |  |  |  |  |  |  |
| ≥100 | 614 (12.2) | 154 (26.1) | 2.54 | 2.08-3.11 | <0.001* | 1.65 | 1.31-2.06 | <0.001* |

Results are presented as n (%). BMI, body mass index; ASA score, American Society of Anesthesiologists score; ECOG-PS, Eastern Cooperative Oncology Group performance status; COPD, chronic obstructive pulmonary disease; CEA, carcinoembryonic antigen; OR, odd ratio; CI, confidence interval
